# Cinnamon: A Multifaceted Medicinal Plant

**DOI:** 10.1155/2014/642942

**Published:** 2014-04-10

**Authors:** Pasupuleti Visweswara Rao, Siew Hua Gan

**Affiliations:** ^1^Faculty of Agro Based Industry, Universiti Malaysia Kelantan, Jeli Campus, Locked Bag No. 100, 17600 Jeli, Kelantan, Malaysia; ^2^Human Genome Centre, School of Medical Sciences, Universiti Sains Malaysia, 16150 Kubang Kerian, Kelantan, Malaysia

## Abstract

Cinnamon (*Cinnamomum zeylanicum*, * and Cinnamon cassia*), the eternal tree of tropical medicine, belongs to the Lauraceae family. Cinnamon is one of the most important spices used daily by people all over the world. Cinnamon primarily contains vital oils and other derivatives, such as cinnamaldehyde, cinnamic acid, and cinnamate. In addition to being an antioxidant, anti-inflammatory, antidiabetic, antimicrobial, anticancer, lipid-lowering, and cardiovascular-disease-lowering compound, cinnamon has also been reported to have activities against neurological disorders, such as Parkinson's and Alzheimer's diseases. This review illustrates the pharmacological prospective of cinnamon and its use in daily life.

## 1. Introduction 

The bark of various cinnamon species is one of the most important and popular spices used worldwide not only for cooking but also in traditional and modern medicines. Overall, approximately 250 species have been identified among the cinnamon genus, with trees being scattered all over the world [1, 2].

Cinnamon is mainly used in the aroma and essence industries due to its fragrance, which can be incorporated into different varieties of foodstuffs, perfumes, and medicinal products [3]. The most important constituents of cinnamon are cinnamaldehyde and* trans*-cinnamaldehyde (Cin), which are present in the essential oil, thus contributing to the fragrance and to the various biological activities observed with cinnamon [4]. A study on* Cinnamomum osmophloeum* (*C*.* osmophloeum*) indicated that the essential oil from cinnamon leaves contains a high level of Cin. Consequently,* C. osmophloeum* is also used as an alternative spice for* C. cassia* [5]. One of the major constituents of essential oil extracted from* C. zeylanicum* named (E)-cinnamaldehyde has an antityrosinase activity [6], while cinnamaldehyde is the principal compound responsible for this activity [7].

Cinnamon bark contains procyanidins and catechins [8]. The components of procyanidins include both procyanidin A-type and B-type linkages [9–11]. These procyanidins extracted from cinnamon and berries also possess antioxidant activities [10, 12].

## 2. Methodology

The current review was conducted using a complete and organized search of the available literature on the medicinal plant cinnamon from 1982 to 2013. The searches were performed using various databases, including PubMed (http://www.ncbi.nlm.nih.gov/pubmed), Science Direct (http://www.sciencedirect.com/), Scopus (http://www.scopus.com/), Scirus (http://www.scirus.com/), and Google Scholar (http://www.scholar.google.com/).

## 3. Traditional Uses

In addition to being used as a spice and flavoring agent, cinnamon is also added to flavor chewing gums due to its mouth refreshing effects and ability to remove bad breath [13]. Cinnamon can also improve the health of the colon, thereby reducing the risk of colon cancer [14].

Cinnamon is a coagulant and prevents bleeding [15]. Cinnamon also increases the blood circulation in the uterus and advances tissue regeneration [16]. This plant plays a vital role as a spice, but its essential oils and other constituents also have important activities, including antimicrobial [17–20], antifungal [21], antioxidant [22–26], and antidiabetic [27–33].

Cinnamon has been used as anti-inflammatory [34–36], antitermitic [36], nematicidal [37, 38], mosquito larvicidal [39], insecticidal [40], antimycotic, [40–43] and anticancer agent [44–47]. Cinnamon has also been traditionally used as tooth powder and to treat toothaches, dental problems, oral microbiota, and bad breath [48, 49].

## 4. Chemical Constituents

Cinnamon consists of a variety of resinous compounds, including cinnamaldehyde, cinnamate, cinnamic acid, and numerous essential oils [50] (Table 1). Singh et al. [51] reported that the spicy taste and fragrance are due to the presence of cinnamaldehyde and occur due to the absorption of oxygen. As cinnamon ages, it darkens in color, improving the resinous compounds [51]. Sangal reported various physiochemical properties of cinnamon (Table 2). The presence of a wide range of essential oils, such as* trans*-cinnamaldehyde, cinnamyl acetate, eugenol, L-borneol, caryophyllene oxide, b-caryophyllene, L-bornyl acetate, E-nerolidol, *α*-cubebene, *α*-terpineol, terpinolene, and *α*-thujene, has been reported [35, 36].

**Figure 1:**
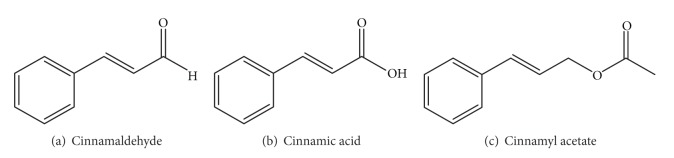
Cinnamyl group-containing compounds.

**Figure 2:**
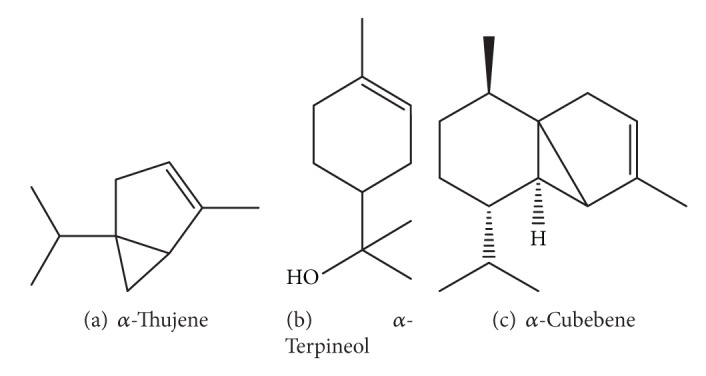
Endocyclic double bond-containing compounds.

**Figure 3:**
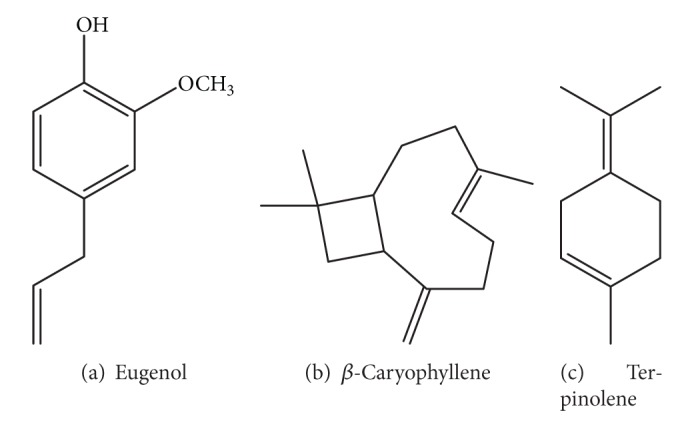
Unconjugated exocyclic double bond-containing compounds.

**Figure 4:**
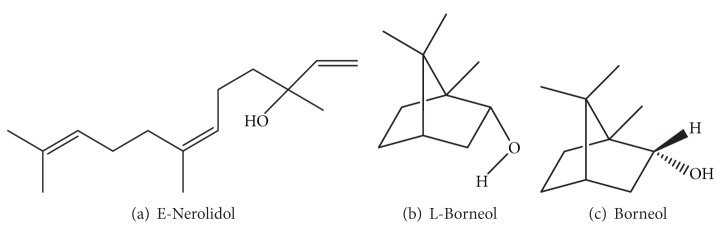
Hydroxy-substituted aliphatic compounds.

**Figure 5:**
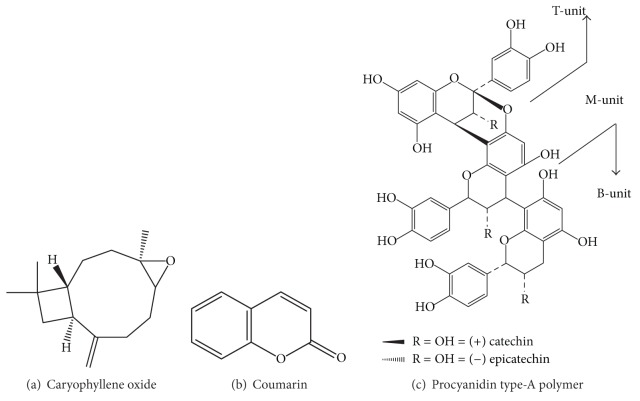
Other miscellaneous compounds containing oxirane, 2-pyranone, and pyran groups.

The chemical structures of some important constituents of cinnamon are shown in Figures 1, 2, 3, 4, and 5.

## 5. Antioxidant Activity

Antioxidant compounds present in foodstuffs play a vital role in human life, acting as health-protecting agents. In addition to this role, antioxidants are one of the key additives used in fats and oils. Even in the food processing industry, antioxidants have been used to delay or prevent food spoilage. Spices and medicinal plants have received rapid consideration as sources of beneficial antioxidants against various diseases [52]. Antioxidants have been considered the most important drivers in the progress and existence of humans, as they respond to free radicals and damage in metabolic diseases and age-related syndromes of humans and other animals [53, 54].

Mancini-Filho et al. reported various extracts of cinnamon, such as ether, aqueous, and methanolic extracts that have shown considerable antioxidant activities [22]. A study on rats reported that the administration of the bark powderof* C. verum* (10%) for 90 days produced antioxidant activities as indicated by cardiac and hepatic antioxidant enzymes, lipid conjugate dienes, and glutathione (GSH) [55]. A research group reported that cinnamon oil potentially exhibits superoxide-dismutase- (SOD-) like activity as indicated by the inhibition of the inhibiting capacity of pyrogallol autoxidation [56].

The aqueous and alcoholic extract (1 : 1) of cinnamon potentially significantly inhibits fatty acid oxidation and lipid peroxidation* in vitro* [23]. Different flavonoids isolated from cinnamon have free-radical-scavenging activities and antioxidant properties [57]. A study of the inhibitory effects of cinnamaldehyde and other compounds of cinnamon on nitric oxide production revealed that cinnamaldehyde possesses potential activity against the production of nitric oxide as well as the expression of inducible nitric oxide. The highest inhibitory activities were reported as 81.5%, 71.7%, and 41.2% at 1.0, 0.5, and 0.1 µg/µL, respectively [58]. Lin et al. reported the* in vivo* antioxidant activity of two different extracts, the ethanolic and hot water extracts of the dry bark of* C. cassia.* The ethanolic extract of* C. cassia* exhibited significant inhibition (96.3%) compared to the natural antioxidant *α*-tocopherol (93.74%) [59]. Overall, cinnamon exhibited higher antioxidant activities compared to that of other dessert spices [60].

The essential oils and some of the major compounds present in cinnamon, including (E)-cinnamaldehyde, eugenol, and linalool, were investigated in reference to peroxynitrite-induced nitration and lipid peroxidation. Eugenol and the essential oils were more effective than the other two compounds [61]. In a comparative study among 26 spices, cinnamon showed the highest antioxidant activity, indicating that it can be applied as an antioxidant used in foods [62]. Another study investigated the effectiveness of a mixture of spices on oxidative stress markers as well as the antioxidant activity in high fructose-fed insulin-resistant rats. The mixture, which consisted of 1 g/100 g cinnamon bark, showed a significant antioxidant activity compared to the fructose alone group [63]. Volatile oils from* C. zeylanicum *showed significant biological activities [64].

Forty-one different volatile compounds in the bark oil of cinnamon have been identified and were found to vary significantly in percentage composition depending on the growth stages and segments of the* C. cassia* tree [65]. To extract essential oil for industrial use, the yields and compositions of bark oil during* Cinnamomum cassia* growth (1–3 years old for the branch bark and 5–12 years old for the stem bark) were determined. These researchers also found that the branch bark fraction tended to yield more essential oil compared to the entire branch, indicating that selecting the bark based on the tree growth stages as well as separating the stem barks into top, center, and lower sections within a tree can significantly improve the extraction efficiency of essential oils.

A preliminary study on* C. malabatrum* leaves was conducted in various types of extracts (n-hexane, alcoholic, and aqueous extracts) to determine the presence of phenolic compounds, which indicate antioxidant activity. All of the extracts had moderate amounts of phenolic compounds and showed potential activity against hydrogen peroxide, nitric oxide, and lipid peroxide free radicals [66]. A recent study investigated the antioxidant properties of several parts (i.e., the leaves, barks, and buds) of* C. cassia*. The ethanolic extract of all of the plant parts had significant antioxidant properties compared with the extraction using the supercritical fluid [67]. The supercritical extracts showed decreased activity compared to the ethanol extracts, indicating that the active components are constituents with high polarity.

*C. tamala* has potential antioxidant activities in diabetic rats [68], while* C. osmophloeum*, a species from Taiwan, has significant* in vitro* and* in vivo* antioxidant activities under oxidative stress [69]. The antioxidant activity of* C. zeylanicum* has been investigated using various methods. In addition to the antioxidant activity, cinnamon can be used as a preservative in cakes and other food products [70]. A recent study reported that pectin film coated with cinnamon leaf extract yielded high antioxidant and antibacterial activities [71]. Dong et al. reported that cinnamaldehyde (E) extracted from* C. cassia* is the main compound and is present in levels as high as 72.7% compared to other volatile components [72]. Cinnamaldehyde (E) is well known for its antityrosinase activity [6, 73]. Currently, much attention is given to tyrosinase inhibitors due to their actions in suppressing hyperpigmentation as well as the unsightly browning effects observed in mushrooms, fruits, and vegetables when they are exposed to sunlight or air. Hence, antityrosinase agents are associated with a wide range of applications, such as cosmetics, medicine, and food [74, 75].

## 6. Anti-Inflammatory Activities

Several studies on medicinal plants and their components have indicated the anti-inflammatory activities of cinnamon [76–81]. Various studies reported the anti-inflammatory activity of cinnamon and its essential oils [34–36]. To date, there are several flavonoid compounds (e.g., gossypin, gnaphalin, hesperidin, hibifolin, hypolaetin, oroxindin, and quercetin) that have been isolated and have anti-inflammatory activities [82–86].

A recent study reported that 2′-hydroxycinnamaldehyde isolated from* C. cassia* bark exhibited an inhibitory effect on the production of nitric oxide by inhibiting the activation of the nuclear factor kappa-light-chain-enhancer of activated B cells (NF-*κ*B), indicating that this substance can potentially be used as an anti-inflammatory agent [87]. The ethanolic extract of* C. cassia* showed significant anti-inflammatory effects by reducing the activation of Src/spleen-tyrosine-kinase- (Src/Syk-) mediated NF-*κ*B [88, 89]. Various compounds contained in* C. ramulus* showed anti-inflammatory effects by suppressing the expression of inducible nitric oxide synthesis (iNOS), cyclooxygenase-2 (COX-2), and nitric oxide (NO) production in the central nervous system (CNS). By this mechanism,* C. ramulus* could be a potential source for the therapeutic treatment or prevention of inflammation-mediated neurodegenerative diseases [90]. Furthermore, the aqueous extract of cinnamon decreases the lipopolysaccharide-induced tumor necrosis factor-*α* levels in the serum [91].

## 7. Neurological Disorders

Cinnamophilin is a novel thromboxane A2 receptor antagonist isolated from* C. philippinensis* [92]. A study reported that cinnamophilin confers protection against ischemic damage in rat brains when administered at 80 mg/kg at different time intervals (2, 4, and 6 h) after insult. The effects were found to have a considerable effect (by 34–43%) on abridged brain infarction [93] and further enhance neurobehavioral outcomes. Cinnamophilin also dramatically condenses the oxygen glucose deprivation-induced neuronal damage in organotypic hippocampal slices in experimental rats. A substance called procyanidin type-A trimer (trimer 1) isolated from cinnamon's water-soluble extract showed that trimer 1 may reduce cell swelling by controlling the movement of intracellular calcium [Ca^2+^]_i_ [94]. Trimer 1 also considerably alleviates the oxygen glucose deprivation-induced diminishing effects on glutamate uptake. The protective effects of trimer 1 in attenuating the diminution in glutamate uptake are possibly arbitrated via their effects on the mitochondria [94].

Parkinson's disease (PD) is the second major widespread neurodegenerative disorder after Alzheimer's disease, with a prevalence of 2% in people 65 years and older [95]. PD protein 7 (PARK7) is an autosomal recessive form of early-onset parkinsonism caused by alterations in the* DJ-1* gene [96]. Khasnavis and Pahan reported that sodium benzoate, a cinnamon metabolite, upregulates* DJ-1* by modulating mevalonate metabolites [97, 98]. Cinnamon and its metabolite sodium benzoate also upregulate the neurotropic factors BDNF (brain-derived neurotropic factors) as well as neurotrophin-3 (NT-3) in the mouse central nervous system [99]. PARK7 is one of the main neuroprotective proteins that protects cells from damage and from the further detrimental effects of oxidative stress; therefore, this protein may be an effective molecule that can be incorporated into the therapeutic intervention of Parkinson's disease [98].

A natural compound isolated from cinnamon extract (CEppt) significantly reduces the formation of toxic *β*-amyloid polypeptide (A*β*) oligomers and prevents its toxicity on neuronal pheochromocytoma (PC12) cells [100]. The study indicated that CEppt resolved the reduced permanence, fully improved deficiencies in locomotion, and totally eradicated the tetrameric species of A*β* in the brain of the fly model of Alzheimer's disease, leading to a noticeable reduction in the 56 kDa A*β* oligomers, reducing plaques and improving the cognitive performance of transgenic mice models [100].

Another study reported that the aqueous extract of* C. zeylanicum *can reduce tau aggregation and filament formation, two of the main features of Alzheimer's disease. The extract can also encourage the complete fragmentation of recombinant tau filaments and cause the considerable modification of the morphology of paired helical filaments from Alzheimer's disease brain [101], indicating the potential of cinnamon in the treatment of Alzheimer's disease.

## 8. Antidiabetic Activity 

A substance from cinnamon has been isolated and coined as “insulin-potentiating factor” (IPF) [102], while the antidiabetic effects of cinnamon bark have been shown in streptozotocin-induced diabetic rats [33]. Several studies have also revealed that cinnamon extracts lower not only blood glucose but also cholesterol levels [103–107].

A study comparing the insulin-potentiating effects of many spices revealed that the aqueous extract of cinnamon was 20-fold higher than the other spices [108]. Methylhydroxychalcone polymer (MHCP) is the purified polymer of hydroxychalcone with the ability to stimulate glucose oxidation [30, 109]. Anderson et al. isolated and characterized the polyphenol type-A polymers from cinnamon and found that these substances act as insulin-like molecules [9]. Following this characterization, a new compound from hydroxycinnamic acid derivatives named naphthalenemethyl ester, which has blood glucose-lowering effects, has been identified [27], further confirming cinnamon's antidiabetic effects.

Several polyphenols have been isolated from cinnamon. These polyphenols include rutin (90.0672%), catechin (1.9%), quercetin (0.172%), kaempferol (0.016%), and isorhamnetin (0.103%) [67, 110]. Cao et al. (2007) demonstrated that the aqueous extract of cinnamon containing polyphenols purified by high performance liquid chromatography (HPLC) showed insulin-like activity [111]. The aqueous extract of cinnamon markedly decreased the absorption of alanine in the rat intestine. Alanine plays a vital role in gluconeogenesis, is altered back to pyruvate in the liver, and is utilized as a substrate for gluconeogenesis [112]. However, another study conducted on diabetic postmenopausal women supplemented with cinnamon showed poor glycemic control [113], even though cinnamon is generally believed to be useful for diabetes. However, it is plausible that differences in the dose of cinnamon used, as well as baseline glucose and lipid levels, have led to these variations.

In a recent study [114], suitable doses of cinnamon (5, 10, and 20 mg/kg) of the linalool chemotype were found to help with glycemic control in diabetics due to enhanced insulin secretion. It is plausible that the amelioration of oxidative stress and the proinflammatory environment in the pancreas may confer protection to pancreatic *β* cells [114], which should be further investigated.

## 9. Antimicrobial Activity

To date, several antimicrobial activities of cinnamon and its oils have been reported in various studies [20, 28, 115]. For example, Matan et al. reported the effects of cinnamon oils on different bacterial (*Pediococcus halophilus *and* Staphylococcus aureus),* fungal* (Aspergillus flavus, Mucor plumbeus, Penicillium roqueforti, *and* Eurotium sp.),* and yeast species (*Candida lipolytica, Pichia membranaefaciens, Debaryomyces hansenii, *and* Zygosaccharomyces rouxii*) [19], indicating that cinnamon is a natural antimicrobial agent.

Goñi et al. described the antibacterial activity of a combination of cinnamon and clove oils against Gram-positive organisms* (Listeria monocytogenes, Enterococcus faecalis, Staphylococcus aureus, *and* Bacillus cereus*), as well as against Gram-negative bacteria (*Salmonella choleraesuis, Escherichia coli, Pseudomonas aeruginosa, *and* Yersinia enterocolitica)* [116]. A study from Hili et al. indicated that cinnamon oils have potential action against various bacteria (*Pseudomonas aeruginosa, Staphylococcus aureus, and Escherichia coli*) and yeast (*Torulopsis utilis, Schizosaccharomyces pombe, Candida albicans, *and* Saccharomyces cerevisiae*) [18]. A recent study reported the activity of the aqueous extract of cinnamon and other plants against oral microflora. Overall, the essential oil from cinnamon is more potent than other tested plant extracts, such as* Azadirachta indica *and* Syzygium aromaticum* [117].

## 10. Anticancer Activity

The aqueous extract and the fraction of cinnamon (procyanidins) from HPLC inhibit vascular endothelial growth factor subtype 2 (VEGFR2) kinase activity, thereby inhibiting the angiogenesis involved in cancer. The results of the study revealed that cinnamon could potentially be used in cancer prevention [44]. Cinnamaldehydes have been synthesized and tested as inhibitors against angiogenesis [118]. Jeong et al. reported that CB403, a chemical that can be synthesized from 2′-hydroxycinnamaldehyde derived from cinnamaldehyde, can inhibit tumor growth. Overall, the antitumor and growth-inhibitory properties of CB403 in animal-based studies as well as in cell culture-based studies indicate the potential of cinnamon to be used as an anticancer agent [119].

Cabello et al. reported that cinnamic aldehyde inhibits the activity of NF-*κ*B and the production of tumor necrosis factor alpha (TNF*α*-) induced interleukin-8 (IL-8) in A375 cells [120]. This inhibition provides additional support to the existing unrecognized role of cinnamic acid as a potential anticancer agent [120]. Fang and others reported the anticancer effect of* trans*-cinnamaldehyde from* C. osmophloeum*, finding that* trans*-cinnamaldehyde showed potential effects in restraining tumor cell growth and in enhancing tumor cell apoptosis [121].

A preliminary study on cinnamon and cardamom against azoxymethane- (AOM-) induced colon cancer in Swiss albino mice has been conducted [122]. Treatments with the aqueous extracts of cinnamon and cardamom augment the activities of the detoxifying and antioxidant enzyme glutathione-s-transferase (GST) with a concomitant reduction in lipid peroxidation levels in animals with colon cancer compared to controls [122]. The essential oils extracted from* C. cassia* inhibit alpha melanocyte-stimulating hormone's induced melanin production, thereby suppressing oxidative stress in murine B16 melanoma cells [7].

## 11. Cardiovascular Diseases

One of the active components isolated from* C. cassia* named 2-methoxycinnamaldehyde (2-MCA) decreases the expression of vascular cell adhesion molecule-1 (VCAM-1) in TNF*α*-activated endothelial cells, suggesting that ischemia/reperfusion (I/R) injury is ameliorated due to the induction of hemeoxygenase- (HO-) 1 [123]. A recent study reported the potential effects of two compounds, cinnamic aldehyde and cinnamic acid, isolated from* C. cassia* against myocardial ischemia [124], indicating that cinnamon also has the potential to be used to treat cardiovascular diseases.

Several studies have reported the protective effects of cinnamaldehyde on the cardiovascular system. Cinnamophilin is one of the important lignans isolated from* C. philippinensis* and has been confirmed to have thromboxane A2 (TXA_2_) receptor blocking activity in rats as well as in guinea pigs [125]. Cinnamophilin acts as a potential thromboxane synthase inhibitor and TXA_2_ receptor antagonist and may be helpful when incorporated in the treatment of diseases involving TXA_2_ disorders [125], such as platelet aggregation [126] and cancers [127]. Cinnamophilin mainly inhibits thromboxane receptor-mediated vascular smooth muscle cell proliferation and may have the potential for use in the prevention of vascular diseases and atherosclerosis [128].

Cinnamaldehyde produces hypotensive effects, which are possibly mainly due to peripheral vasodilatation in anesthetized dogs and guinea pigs [129]. The vasodilatation induced by cinnamaldehyde in dogs lasted and remained over the recovery period of the fall in blood pressure to the baseline [130]. A recent study showed that cinnamaldehyde expands rat vascular smooth muscle in an endothelium-independent manner. The ability of cinnamaldehyde in vasodilatory function may be because it impedes both Ca^2+^ influx and Ca^2+^ release [131]. Cinnamaldehyde averts the progress of hypertension in types 1 and 2 diabetes by abridging vascular contractility, in addition to its insulinotropic effect in insulin deficiency [132].

## 12. Cholesterol- and Lipid-Lowering Effects

The administration of cinnamon to mice positively affected the lipid profile, whereby the high density lipoprotein (HDL) cholesterol levels decreased, and plasma triglycerides were reduced [27]. Another study by [133] found a reduction in the total cholesterol, triglycerides, and low-density lipoproteins in rats administered* Cinnamomum cassia *powder (15%) for 35 days. Additionally, cinnamon oils reduced the cholesterol levels in broiler chickens [134]. A study by Khan et al. reported that the administration of cinnamon at 1, 3, and 6 g doses per day caused a reduction in serum glucose, triglyceride, total cholesterol, and LDL cholesterol levels in humans [104].

## 13. Advanced Glycation End Products (AGEs)

Different types of phenolic and flavonoid compounds have been isolated from cinnamon. Epicatechin, catechin, and procyanidin B2, which are the phenolic compounds isolated from cinnamon, showed noteworthy and potentially inhibitory activities on the formation of AGEs. These antiglycation activities of the phenolic compounds not only are attributed to their antioxidant activities but also are associated with the entrapping capabilities of reactive carbonyl species, such as methylglyoxal (MGO), an intermediate reactive carbonyl of AGE formation [10, 135]. The inhibition of AGE formation by trapping the reactive carbonyl species could be a logical therapeutic approach to treat diabetes and its complications [10].

## 14. Conclusions

Cinnamon has been used as a spice in daily life without any side effects. Several reports have dealt with the numerous properties of cinnamon in the forms of bark, essential oils, bark powder, phenolic compounds, flavonoids, and isolated components. Each of these properties plays a key role in the advancement of human health. The antioxidant and antimicrobial activities may occur through the direct action on oxidants or microbes, whereas the anti-inflammatory, anticancer, and antidiabetic activities occur indirectly via receptor-mediated mechanisms. The significant health benefits of numerous types of cinnamon have been explored. Further investigations are necessary to provide additional clinical evidence for the traditional uses of this spice against cancer and inflammatory, cardioprotective, and neurological disorders.

## Figures and Tables

**Table 1:** Chemical constituents of different parts of cinnamon [136] (Vangalapati et al., 2012 [2]).

Part of the plant	Compound
Leaves	Cinnamaldehyde: 1.00 to 5.00%
	Eugenol: 70.00 to 95.00%

Bark	Cinnamaldehyde: 65.00 to 80.00%
	Eugenol: 5.00 to 10.00%

Root bark	Camphor: 60.00%

Fruit	*trans*-Cinnamyl acetate (42.00 to 54.00%) and caryophyllene (9.00 to 14.00%)

*C. zeylanicum *buds	Terpene hydrocarbons: 78.00%
	*alpha*-Bergamotene: 27.38%
	*alpha*-Copaene: 23.05%
	Oxygenated terpenoids: 9.00%

*C. zeylanicum *flowers	(E)-Cinnamyl acetate: 41.98%
	*trans-alpha*-Bergamotene: 7.97%
	Caryophyllene oxide: 7.20%

**Table 2:** Physicochemical properties of cinnamon(Sangal., 2011 [1]).

Parameter	Leaf oil	Bark oil
Specific gravity (20°C)	1.030–1.050	1.010–1.030
Optical rotation (°) (20°C)	1°96′–0°40′	Slightly laevorotatory
Refractive index (20°C)	1.529–1.537	1.573–1.591
Aldehyde content	4%	65–76%
Eugenol content	77.3–90.5%	4–10%
Solubility characteristics	Soluble in 1.5 volumes of 70% alcohol	Soluble in 2.0–3.0 volumes of 70% alcohol

## Abbreviations

GSH: Glutathione
SOD: Superoxide dismutase
Src/Syk: Src/spleen tyrosine kinase
iNOS: Inducible nitric oxide synthesis
COX-2: Cyclooxygenase-2
NO: Nitric oxide
CNS: Central nervous system
PARK7: PD protein 7
NT-3: Neurotrophin-3
A*β*: *β*-Amyloid polypeptide
PC 12: Pheochromocytoma 12
IPF: Insulin-potentiating factor
MHCP: Methylhydroxychalcone polymer
VEGFR2: Vascular endothelial growth factor subtype 2
TNF*α*: Tumor necrosis factor-alpha interleukin-8 (IL-8)
AOM: Azoxymethane
GST: Glutathione-s-transferase
2-MCA: 2-Methoxycinnamaldehyde
VCAM-1: Vascular cell adhesion molecule-1
HO: Heme oxygenase (HO)
TXA_2_: Thromboxane A2
HDL cholesterol: High-density lipoprotein cholesterol
MGO: Methylglyoxal.
